# Supplementary material for: Development of an Instrument to Evaluate the Intake of Liquids, Food and Supplements in Endurance Competitions: Nutritional Intake Questionnaire for Endurance Competitions—NIQEC
Source: Nutrients. 2023 Apr 19;15(8):1969. doi: 10.3390/nu15081969 (PMC10143029; doi:10.3390/nu15081969)
Supplement: Supplementary file 1 [file nutrients-15-01969-s001.zip › File S1-Nutritional Intake Questionnaire for Endurance Competitions - NIQEC.pdf]

# Cuestionario sobre Ingesta Nutricional para Competiciones de Resistencia (NIQEC).

Las respuestas a este cuestionario servirán para valorar y cuantificar el consumo de líquidos, alimentos y suplementos durante la competición. Los resultados colectivos del cuestionario nos ayudarán a plantear recomendaciones para mejorar la tolerancia de la ingesta y disminuir la aparición de problemas gastrointestinales durante las competiciones.

Se trata de un cuestionario CONFIDENCIAL. Toda la información será analizada por personal técnico sujeto al secreto profesional y utilizada exclusivamente para los fines descritos. La Ley Orgánica 3/2018 de Protección de Datos será respetada en todo momento. Este estudio ha sido aprobado por el comité de ética de la Universidad de Alicante (#UA 2021-02-01). En el informe de resultados no podrán ser identificadas las respuestas de ninguna persona de forma individualizada.

Para participar en el estudio es necesario ser mayor de edad y contestar INDIVIDUALMENTE. Por ello te pedimos que respondas siguiendo las instrucciones de cada pregunta para contestar. Es muy importante que contestes TODAS LAS PREGUNTAS ya que muchas se tratan conjuntamente, de manera que si dejas alguna pregunta sin contestar es probable que otras que hayas contestado se anulen y se pierda la información para el análisis de resultados.

Para llevar a cabo esta investigación se ha creado un Grupo de Trabajo formado: Rubén Jiménez (rja10@gcloud.ua.es), Isabel Sospedra (isospedra@ua.es) y José Miguel Martínez (josemiguel.ms@ua.es). Si necesitas realizar alguna consulta te puedes dirigir a cualquiera de sus miembros.

MUCHAS GRACIAS POR TU COLABORACIÓN

CONSENTIMIENTO:
- Soy mayor de edad.
- He sido informado/a de las características del Proyecto de Investigación titulado: “Estudio del consumo de líquidos, alimentos y suplementos durante competiciones de resistencia de larga duración”.
- He leído la información del proyecto, y he podido formular las dudas que me han surgido al respecto.
- Considero que he entendido dicha información.
- Estoy informado/a de la posibilidad de retirarme en cualquier momento del estudio.
- Estoy informado del modo en que serán tratados mis datos.

*Obligatorio

1. En virtud de tales condiciones ¿consientes participar en este estudio? *

   *Marca solo un óvalo.*

   - Si
   - No

*Salta a la pregunta 2*

DATOS SOCIODEMOGRÁFICOS E IDENTIFICATIVOS

2. 1. Número de dorsal

   Indica el nº de dorsal que te ha adjudicado/asignado la organización para realizar esta competición.

3. 2. Denominación de la competición y/o distancia de la prueba realizada en km

   Indicar el nombre (denominación) y el número de km de los que se compone la competición (por ejemplo: Maratón, 42.2km)

4. 3. Tiempo de finalización de la prueba

   Indicar horas, minutos y segundos (resultado oficial). Por ejemplo: 07:32:48.

   *Ejemplo: 8:30 a.m.*

5. 4. En caso de no haber finalizado la prueba, indique los motivos

6. 5. Sexo

*Marca solo un óvalo.*

- Hombre
- Mujer

7. 6. Fecha de nacimiento

_______________________________

*Ejemplo: 7 de enero del 2019*

8. 7. Edad actual en años

*Marca solo un óvalo.*

- 18
- 19
- 20
- 21
- 22
- 23
- 24
- 25
- 26
- 27
- 28
- 29
- 30
- 31
- 32
- 33
- 34
- 35
- 36
- 37
- 38
- 39
- 40
- 41
- 42
- 43
- 44
- 45
- 46
- 47
- 48
- 49
- 50

- 51
- 52
- 53
- 54
- 55
- 56
- 57
- 58
- 59
- 60
- 61
- 62
- 63
- 64
- 65
- 66
- 67
- 68
- 69
- 70
- 71
- 72
- 73
- 74
- 75

9. 8. Comunidad Autónoma de residencia

*Marca solo un óvalo.*

- Andalucía
- Cataluña
- Comunidad de Madrid
- Comunidad Valenciana
- Galicia
- Castilla y León
- País Vasco
- Canarias
- Castilla-La Mancha
- Región de Murcia
- Aragón
- Isla Baleares
- Extremadura
- Principado de Asturias
- Navarra
- Cantabria
- La Rioja
- Melilla
- Ceuta

10. 9. Estatura

Indicar valor en cm (ejemplo 175 ó 160.5)

_______________________

11. 10. Peso actual

Indicar valor en kg (ejemplo 80 ó 65.7)

_______________________

*Salta a la pregunta 12*

DATOS DEPORTIVOS

12. 11. ¿Estás federado/a actualmente?

*Marca solo un óvalo.*

- Sí
- No

13. 12. Principalmente, ¿a qué nivel compites?

*Marca solo un óvalo.*

- Regional
- Nacional
- Internacional

14. 13. Actualmente, ¿en que modalidad deportiva compites principalmente ?

*Marca solo un óvalo.*

- Ciclismo de carretera
- Ciclismo de montaña (maratón o ultramaratón)
- Natación (aguas abiertas)
- Atletismo (media maratón, maratón o ultramaratón)
- Carreras de montaña
- Duatlón
- Triatlón
- Otro: ______________________

15. 14. ¿Cuántos años llevas realizando dicha modalidad deportiva?

Seleccionar número de años aproximados

*Marca solo un óvalo.*

- 1
- 2
- 3
- 4
- 5
- 6
- 7
- 8
- 9
- 10
- 11
- 12
- 13
- 14
- 15
- 16
- 17
- 18
- 19
- 20
- 21
- 22
- 23
- 24
- 25
- 26
- 27
- 28
- 29
- 30
- 31
- 32

- 33
- 34
- 35
- 36
- 37
- 38
- 39
- 40

16. 15. Número de sesiones de entrenamiento semanal de manera habitual

Si has realizado un entrenamiento de natación por la mañana y otro entrenamiento de fuerza por la tarde, se consideran dos sesiones de entrenamiento

*Marca solo un óvalo.*

- 1 vez por semana
- 2 veces por semana
- 3 veces por semana
- 4 veces por semana
- 5 veces por semana
- 6 veces por semana
- 7 veces por semana
- 8 veces por semana
- más de 8 veces por semana

17. 16. ¿Realizas 2 o más sesiones de entrenamiento algún día a la semana de manera habitual?

Ten en cuenta lo indicado en la pregunta anterior sobre número de sesiones de entrenamiento semanal

*Marca solo un óvalo.*

- Sí
- No

18. 17. ¿Cuántas horas entrenas a la semana de manera habitual?

Seleccionar horas totales aproximadas

*Marca solo un óvalo.*

- 1
- 2
- 3
- 4
- 5
- 6
- 7
- 8
- 9
- 10
- 11
- 12
- 13
- 14
- 15
- 16
- 17
- 18
- 19
- 20
- 21
- 22
- 23
- 24
- 25
- 26
- 27
- 28
- 29
- 30
- 31
- 32

- 33
- 34
- 35
- 36
- 37
- 38
- 39
- 40

*Salta a la pregunta 19*

INGESTA DE LÍQUIDOS, ALIMENTOS Y SUPLEMENTOS EN LA HORA PREVIA A LA COMPETICIÓN.

19. 18. Detalla las bebidas y la cantidad, que hayas consumido en la hora previa a la competición.

Detallar nombre y marca comercial.
Ejemplos: 500 ml de agua y una taza de café solo (indica si se les ha añadido azúcar, leche, bebidas vegetales,…); 1 cacito de Iso-Lyte Sport Drink de Amix en 500ml de agua.

20. 19. Detalla los alimentos y la cantidad, que hayas consumido en la hora previa a la competición.

Ejemplo: 1 plátano mediano y 50 g de dulce de membrillo

21. 20. Detalla los suplementos y la cantidad que hayas consumido en la hora previa a la competición (barritas, geles, cafeína, BCAA, nitratos, creatina...).

Detallar nombre y marca comercial.
Ejemplos: 1 cacito de monohidrato de creatina marca My protein; 1 energy bar de Crown Sport Nutrition.

INGESTA DE LÍQUIDOS, ALIMENTOS Y SUPLEMENTOS DURANTE LA COMPETICIÓN.

22. 21. ¿Has consumido bebidas durante la competición? (tipo agua, bebidas isotónicas, refrescos, infusiones...)

*Marca solo un óvalo.*

- Sí
- No

23. 22. Agua

Indicar cantidad total de agua ingerida en la prueba, sin contabilizar el agua mezclada con suplementos u otro alimentos.
Ejemplos: 1'5 litros; 2 bidones de 500 ml

24. 23. Bebidas isotónicas

Detallar nombre, marca y cantidad total ingerida durante la prueba.
Ejemplos: 50 g de Carbolider Xtreme Fullgas en 1L de agua; 3 botes de 500ml de IsoPowder Limón de NutriSport, ...

25. 24. Café o infusiones

Indicar cantidad total de café o infusiones ingeridas durante la prueba.
Ejemplo: 3 vasos de 200ml de café

26. 25. Caldos o sopas

Indicar cantidad total de caldos o sopas ingeridas durante la prueba. Ejemplo: 2 vasos de caldo de verduras de 200ml

27. 26. Refrescos

Indicar cantidad total de refrescos ingeridos durante la prueba. Ejemplos: 500 ml de Coca-cola; 3 vasos de 200 ml de Aquarius.

28. 27. ¿Has consumido alimentos sólidos o semisólidos durante la competición?

Ejemplos: barritas, fruta, sándwiches, gominolas,...

*Marca solo un óvalo.*

- Sí
- No

29. 28. Consumo de fruta fresca o desecada.

Indicar cuáles, cuántas y el peso aproximado. Ejemplos: Un plátano (150 g) y media naranja (100 g), 1 puñado de uvas pasas (30 g)

30. 29. Consumo de frutos secos.

Indicar cuáles y la cantidad aproximada (peso o número).
Ejemplos: 50 g de nueces o 10 almendras

31. 30. Consumo de barritas comerciales.

Indicar nombre, marca comercial y cantidad consumida (nº de unidades).
Ejemplo: 3 barritas energy bar de Crown Sport Nutrition.

32. 31. Consumo de barritas caseras.

Indicar ingredientes, peso de cada ingrediente y unidades consumidas.
Ejemplo: 1 barrita con 30g de avena, 3 dátiles y medio plátano

33. 32. Consumo de otros alimentos.

Indicar ingredientes y cantidad (peso).
Ejemplos: Sándwich de pavo con 2 rebanadas de pan de molde y 30 g de pavo.

34. 33. ¿Has consumido geles durante la competición?

*Marca solo un óvalo.*

- Sí
- No

35. 34. ¿Cuántos geles has consumido?

Indicar número de unidades consumidas durante la prueba.

36. 35. Especifica tipo de gel.

Detallar nombre, marca y peso.
Ejemplo: 3 Ejemplos: geles 226ERS Energy Bio Cola de 25 g

37. 36. ¿Has consumido algún tipo de suplemento no incluido en las cuestiones anteriores durante la competición? (Pastillas de sales, cafeína, BCAA,…)

*Marca solo un óvalo.*

- Sí
- No

38. 37. ¿Cuál o cuáles has tomado?

Detallar nombre, marca y cantidad total ingerida durante la prueba.
Ejemplo: 2 cápsulas de cafeína de 100mg de HSN

INGESTA DE LÍQUIDOS, ALIMENTOS Y SUPLEMENTOS EN LA HORA POSTERIOR A LA COMPETICIÓN.

39. 38. Detalla las bebidas y cantidad, que hayas consumido en la hora posterior a la finalización de la competición.

Detallar nombre y marca.
Ejemplos: 200ml de agua y una taza de café solo (indicar si se les ha añadido azúcar, leche, bebidas vegetales,...); 500ml de Powerade

40. 39. Detalla los alimentos y cantidad que hayas consumido en la hora posterior a la finalización de la competición.

Detallar nombre y marca.
Ejemplo: 1 plátano mediano y 50 g de dulce de membrillo.

41. 40. Detalla los suplementos y cantidad que hayas consumido en la hora posterior a la finalización de la competición (batido recuperador, proteínas, geles, barritas, BCAA, creatina...).

Detallar nombre y marca.
Ejemplo: 1 cacito de 226ERS RECOVERY DRINK batido recuperador

*Salta a la pregunta 42*

PROBLEMAS GASTROINTESTINALES

42. 41. ¿Está diagnosticado de alguna alergia o intolerancia alimentaria?

*Marca solo un óvalo.*

- Sí
- No

43. 42. En caso afirmativo indicar cuál.

_______________________________

44. 43. ¿Has sufrido algún tipo de problema o malestar gastrointestinal durante la competición?

*Marca solo un óvalo.*

- Sí
- No

45. 44. Señala el tipo de problema o malestar gastrointestinal y la intensidad de los síntomas.

Indica del 1 al 10 la gravedad/intensidad, siendo 1 "Sin síntomas"; 5 “Síntomas moderados”; y 10 “Síntomas severos”.

*Marca solo un óvalo por fila.*

| | 1 | 2 | 3 | 4 | 5 | 6 | 7 | 8 |
|---|---|---|---|---|---|---|---|---|
| Eructos | | | | | | | | |
| Acidez | | | | | | | | |
| Reflujo | | | | | | | | |
| Hinchazón | | | | | | | | |
| Gases | | | | | | | | |
| Mareos | | | | | | | | |
| Náuseas | | | | | | | | |
| Vómitos | | | | | | | | |
| Flato | | | | | | | | |
| Dolor de Estómago | | | | | | | | |
| Dolor intestinal izquierdo | | | | | | | | |
| Dolor intestinal derecho | | | | | | | | |
| Ganas de defecar | | | | | | | | |
| Defecación: consistencia normal | | | | | | | | |
| Defecación: anormal heces blandas | | | | | | | | |
| Defecación: diarrea | | | | | | | | |
| Defecación: | | | | | | | | |

Defecación: heces con sangre

46. 45. ¿Crees que el problema o malestar gastrointestinal sufrido durante la competición se relaciona con el consumo de algún alimento, líquido o suplemento?

*Marca solo un óvalo.*

- Sí
- No

47. 46. En caso afirmativo, indicar con qué líquido, alimento o suplemento lo relacionas.

En caso de otros, indicar cuales.

*Selecciona todos los que correspondan.*

- [ ] Bebidas isotónicas
- [ ] Agua
- [ ] Refrescos
- [ ] Té o infusión
- [ ] Caldos o sopas
- [ ] Sándwich o bocadillo
- [ ] Barritas caseras
- [ ] Fruta
- [ ] Fruta deshidratada
- [ ] Frutos secos
- [ ] Geles
- [ ] Barritas comerciales
- [ ] Gominolas
- [ ] Suplementos de cafeína
- [ ] Otros

48. 47. Explica los motivos por los que crees que ha podido ocurrir:

_______________________________________________

_______________________________________________

_______________________________________________

_______________________________________________

_______________________________________________

*Salta a la pregunta 49*

PLANIFICACIÓN DE LA PRUEBA A NIVEL DIETÉTICO-NUTRICIONAL

49. 48. ¿Has realizado una preparación especial para esta competición?

Sesiones de entrenamiento a nivel gastro-Intestinal, dieta específica días previos,...

*Marca solo un óvalo.*

- Sí
- No

50. 49. ¿Planificaste la estrategia dietético-nutricional para la competición?

Ejemplos: revisar el perfil de la prueba, los avituallamientos, preparar los líquidos, alimentos y suplementos que vas a tomar por hora o cada ciertos km

*Marca solo un óvalo.*

- Sí
- No

51. 50. ¿Has recibido asesoramiento nutricional para realizar la prueba?

*Selecciona todos los que correspondan.*

- [ ] Ninguno
- [ ] Dietista-nutricionista
- [ ] Médico
- [ ] Entrenador
- [ ] Enfermero
- [ ] Farmacéutico
- [ ] Fisioterapeuta
- [ ] Amigo/familiar
- [ ] Sitio web/internet
- [ ] Otro: ____________________

Este contenido no ha sido creado ni aprobado por Google.

Google Formularios
