# Supplementary material for: Development of an Instrument to Evaluate the Intake of Liquids, Food and Supplements in Endurance Competitions: Nutritional Intake Questionnaire for Endurance Competitions—NIQEC
Source: Nutrients. 2023 Apr 19;15(8):1969. doi: 10.3390/nu15081969 (PMC10143029; doi:10.3390/nu15081969)
Supplement: Supplementary file 1 [file nutrients-15-01969-s001.zip › Supplementary material section NIQEC.pdf]

Table S1: Sociodemographic data of accredited D-N and athletes specialized in endurance sports participating in phases 1 and 3 (Focus Groups and Delphi survey).

Accredited D-N			Athletes		
Demographic data	Sex	Years of experience	Demographic data	Sex	Years of experience
Participant 1	Woman	4	Participant 1	Man	8
Participant 2	Man	5	Participant 2	Woman	15
Participant 3	Man	2	Participant 3	Man	5
Participant 4	Man	6	Participant 4	Woman	8
Participant 5	Woman	9	Participant 5	Man	18
Participant 6	Man	4	Participant 6	Man	10
Participant 7	Man	8	Participant 7	Woman	7
Participant 8	Man	5	Participant 8	Man	5
Participant 9	Woman	10	Participant 9	Woman	4
Participant 10	Man	7	Participant 10	Man	7
Participant 11	Woman	12	Participant 11	Man	4
Participant 12	Woman	5	Participant 12	Man	6
Participant 13	Man	4	Participant 13	Man	15
Participant 14	Man	8	Participant 14	Woman	25
Participant 15	Man	11	Participant 15	Man	25
Participant 16	Man	6			
Participant 17	Man	12			

Table S2. Map of codes and coherencies of demographic data for the proposal for online questionnaire validation for endurance events.

RESEARCH QUESTIONS	THEME	METACODES	CODES	SUBCODES
What questions would you ask to find out the sociodemographic data of the athlete?	A. Sociodemographic data	A1. Questions referring to demographic data from the point of view of professional dietitians-nutritionists	A1.1 Sex	A 1.1.1 Woman
				A 1.1.2 Man
			A1.2 Birthdate	A 1.2 Day, month and year number
			A1.3 Age	A 1.3 Years in number
			A1.4 Autonomous Community of residence	A 1.4.1 Andalusía
				A.1.4.2 Catalonia
				A.1.4.3 Community of Madrid
				A.1.4.4 Valencian Community
				A.1.4.5 Galician
				A.1.4.6 Castile and Leon
				A.1.4.7 Basque Country
				A.1.4.8 Canary Islands
				A.1.4.9 Castilla La Mancha
				A.1.4.10 Region of Murcia
				A.1.4.11 Aragon
				A.1.4.12 Balearic Islands
				A.1.4.13 Estremadura
				A.1.4.14 Principality of Asturias
				A.1.4.15 Navarre
				A.1.4.16 Cantabria
				A.1.4.17 La Rioja
				A.1.4.18 Melilla
				A.1.4.19 Ceuta
			A1.5 Height	A.1.5 Centimetres in numbers
			A1.6 Current Weight	A.1.6 Kilograms in numbers
		A.2. Questions referring to demographic data from the point of view of the athletes	A2.1 Sex	A 2.1.1 Woman
				A 2.1.2 Man
			A2.2 Birthdate	A 2.2 Day, month and year number
			A2.3 Age	A 2.3 Número en Años
			A2.4 Autonomous Community of residence	A 2.3 Years in number
				A 2.4.1 Andalusía
				A.2.4.2 Catalonia
				A.2.4.3 Community of Madrid
				A.2.4.4 Valencian Community
				A.2.4.5 Galician
				A.2.4.6 Castile and Leon
				A.2.4.7 Basque Country
				A.2.4.8 Canary Islands
				A.2.4.9 Castilla La Mancha

				A.2.4.10 Region of Murcia
				A.2.4.11 Aragon
				A.2.4.12 Balearic Islands
				A.2.4.13 Estremadura
				A.2.4.14 Principality of Asturias
				A.2.4.15 Navarre
				A.2.4.16 Cantabria
				A.2.4.17 La Rioja
				A.2.4.18 Melilla
			A2.5 Height	A.2.4.19 Ceuta
			A2.6 Current Weight	A.2.5 Centimetres in numbers

Table S3. Map of codes and coherencies of Sports data for the proposal for online questionnaire validation for endurance events.

RESEARCH QUESTIONS	THEME	METACODES	CODES	SUBCODES
What questions would you ask to find out the sports data of the athletes?	B. Sports data	B1. Questions referring to sports data from the point of view of expert dietitians-nutritionists	B1.1 Main competition sport	B1.1.1 Mountain biking or MTB
				B1.1.2 Open-water swimming
				B1.1.3 Half marathon
				B1.1.4 Marathon
				B1.1.5 Ultramarathon
				B1.1.6 Mountain running
				B1.1.7 Duathlon
				B1.1.8 Triathlon
			B1.2 Number of training sessions per week	B1.2.1 1 time per week
				B1.2.2 2 times per week
				B1.2.3 3 times per week
				B1.2.4 4 times per week
				B1.2.5 5 times per week
				B1.2.6 6 times per week
				B1.2.7 7 times per week
				B1.2.8 8 times per week
				B1.2.9 >8 times per week
			B1.3 Performing 2 or more training sessions per day	B1.3.1 Yes
				B1.3.2 No
			B 1.4 Hours of training per week	B1.4.1 Hours in numbers
			B 1.5 Years practicing the sport	B 1.5.1 Years in numbers
			B1.6 Federated	B1.6.1 Yes
				B1.6.2 No
			B1.7 Competition level	B1.7.1 National
				B1.7.2 International
			B1.8 Race number	B.1.8.1 Race number in the competition
			B1.9 Distance of the event performed	B1.9.1 Kilometers in numbers
			B1.10 Event completion time	B1.10.1 Hours, minutes and seconds in numbers
			B1.11 In case you haven't finished the event	B1.11.1 Motives or reasons in case of not having finished the test
		B2. Questions referring to sports data from the point of view of the athletes	B2.1 Main competition sport	B2.1.1 Mountain biking or MTB
				B2.1.2 Open-water swimming
				B2.1.3 Half marathon
				B2.1.4 Marathon
				B2.1.5 Ultramarathon
				B2.1.6 Mountain running
				B2.1.7 Duathlon
				B2.1.8 Triathlon
			B2.2 Number of training sessions per week	B2.2.1 1 time per week

				B2.2.2 2 times per week
				B2.2.3 3 times per week
				B2.2.4 4 times per week
				B2.2.5 5 times per week
				B2.2.6 6 times per week
				B2.2.7 7 times per week
				B2.2.8 8 times per week
				B2.2.9 >8 times per week
			B2.3 Performing 2 or more training sessions per day	B2.3.1 Yes
				B2.3.2 No
			B2.4 Hours of training per week	B2.4.1 Hours in numbers
			B2.5 Years practicing the sport	B2.5.1 Years in numbers
			B2.6 Federated	B2.6.1 Yes
				B2.6.2 No
			B2.7 Competition level	B2.7.1 National
				B2.7.2 International
			B2.8 Race number	B2.8.1 Race number in the competition
			B2.9 Distance of the event performed	B2.9.1 Kilometers in numbers
			B2.10 Event completion time	B2.10.1 Hours, minutes and seconds in numbers
			B2.11 In case you haven't finished the event	B2.11.1 Motives or reasons in case of not having finished the test

Table S4. Map of codes and coherencies of Consumption of food, liquids and supplements during the hour before, during and in the hour after the competition for the proposal for online questionnaire validation for endurance events.

RESEARCH QUESTIONS	THEME	METACODES	CODES	SUBCODES
What questions would you ask to know the consumption of food, liquids and supplements before, during and after a competition?	C. Consumption of food, liquids and supplements, before, during and after the competition	C1. Questions referring to consumption of food, liquids and supplements 1 hour before the competition from the point of view of expert dietitians-nutritionists	C1.1 Solid food	C1.1.1 Details of the name, quantity, size and brand of the food or supplement consumed.
			C1.2 Liquids	C1.2.1 Detail of amount consumed and brand in case of commercial drink
			C1.3 Supplements	C1.3.1 Detail of the supplement consumed specifying quantity and brand
		C2. Questions referring to consumption of food, liquids and supplements during the competition from the point of view of expert dietitians-nutritionists	C2.1 Consumption of solid/semi-solid food during the competition	C.2.1.1 Yes
				C.21.2 No
			C2.2 Solid food	C2.1.1 Fruits
				C2.1.2 Dried fruits
				C2.1.3 Homemade energy bars
				C2.1.4 Commercial energy bars or commercial Jellys
				C2.1.5 Others
			C2.3 Gels consumption during the competition	C2.3.1 Yes
				C2.3.2 No
			C2.4 Quantity of gels consumed during the competition	C2.4 Units number
			C2.5 Type of gel	C2.5.1 Detail of the name of the product, brand and quantity
			C2.6 Drinks consumption during the competition	C2.6.1 Yes
				C2.6.2 No
			C2.7 Drinks	C2.7.1 Water
				C2.7.2 Refreshment
				C2.7.3 Coffee/Infusions
				C2.7.4 Soups
				C2.7.5 Replacement drinks (or isotonic of for athletes)
		C3. Questions referring to Consumption of food, liquids and supplements 1 hour after the competition from the point of view of expert dietitians-nutritionists	C3.1 Solid food	C3.1.1 Details of the name, quantity, size and brand of the food or supplement consumed.
			C3.2 Liquids	C3.2.1 Detail of amount consumed and brand in case of commercial drink
			C3.3 Supplements	C3.3.1 Detail of the supplement consumed specifying quantity and brand

		C4. Questions referring to consumption of food, liquids and supplements 1 hour before the competition from the point of view of the athletes	C4.1 Solid food	C4.1.1 Details of the name, quantity, size and brand of the food or supplement consumed.
			C4.2 Liquids	C4.2.1 Detail of amount consumed and brand in case of commercial drink
			C4.3 Supplements	C4.3.1 Detail of the supplement consumed specifying quantity and brand
		C5. Questions referring to consumption of food, liquids and supplements during the competition from the point of view of the athletes	C5.1 Consumption of solid/semi-solid food during the competition	C.5.1.1 Yes
				C.51.2 No
			C5.2 Solid food	C5.2.1 Fruits
				C5.2.2 Dried fruits
				C5.2.3 Homemade energy bars
				C5.2.4 Commercial energy bars or commercial Jellys
				C5.2.5 Others
			C5.3 Gels consumption during the competition	C5.3.1 Yes
				C5.3.2 No
			C5.4 Quantity of gels consumed during the competition	C5.4 Units number
			C5.5 Type of gel	C5.5.1 Detail of the name of the product, brand and quantity
			C5.6 Drinks consumption during the competition	C5.6.1 Yes
				C5.6.2 No
			C5.7 Drinks	C5.7.1 Water
				C5.7.2 Refreshment
				C5.7.3 Coffee/Infusions
				C5.7.4 Soups
				C5.7.5 Replacement drinks (or isotonic of for athletes)
		C6. Questions referring to consumption of food, liquids and supplements 1 hour after the competition from the point of view of the athletes	C6.1 Solid food	C6.1.1 Details of the name, quantity, size and brand of the food or supplement consumed.
			C6.2 Liquids	C6.2.1 Detail of amount consumed and brand in case of commercial drink
			C6.3 Supplements	C6.3.1 Detail of the supplement consumed specifying quantity and brand

Table S5. Map of codes and coherencies of Gastrointestinal complaints for the proposal for online questionnaire validation for endurance events.

RESEARCH QUESTIONS	THEME	METACODES	CODES	SUBCODES
What questions would you ask to find out about the gastrointestinal complaints that can occur in a competition?	D. Gastrointestinal complaints	D1. Questions referring to castrointestinal complaints from the point of view of expert dietitians-nutritionists	D1.1 Diagnosis of any food allergy or intolerance	D1.1.1 Yes
				D1.1.2 No
			D1.2 Allergy or food intolerance	D1.2.1 Name of allergy or food intolerance
			D1.3 Gastrointestinal discomfort or complaint during the test	D1.3.1 Yes
				D1.3.2 No
			D1.4 Type of discomfort	D1.4.1 Belch
				D1.4.2 Heartburn
				D1.4.3 Swelling
				D1.4.4 Stomachache
				D1.4.5 Urge to regurgitate
				D1.4.6 Regurgitation
				D1.4.7 Gases
				D1.4.8 Vomits
				D1.4.9 Urge to defecate
				D1.4.10 Left intestinal pain
				D1.4.11 Right intestinal pain
				D1.4.12 Defecation: Normal consistency
				D1.4.13 Defecation: abnormal loose feces
				D1.4.14 Defecation: diarrhea
				D1.4.15 Defecation: bloody feces
				D1.4.16 Nausea
				D1.4.17 Dizziness
				D1.4.18 Acute transient abdominal pain
			D1.5 Gastrointestinal discomfort/problem during the test is related to the consumption of any food, liquid or supplement	D1.5.1Yes
				D1.5.2 No
			D1.6 In case of discomfort, the type of food that causes it En caso de presentar malestar, tipo de alimento que lo ocasiona.	D1.6.1 Examples of foods that can cause gastro-intestinal discomfort/problems
			D1.7 Reasons or reasons why it could have happened	D1.7.1 Explanation and detail of the reasons or reasons why it could have happened
		D2. Questions referring to gastrointestinal complaints from the point of view of athletes	D2.1 Diagnosis of any food allergy or intolerance	D2.1.1 Yes
				D2.1.2 No
			D2.2 Allergy or food intolerance	D2.2.1 Name of allergy or food intolerance
			D2.3 Gastrointestinal discomfort or complaint during the test	D2.3.1 Yes
				D2.3.2 No
			D2.4 Type of discomfort	D2.4.1 Belch
				D2.4.2 Heartburn
				D2.4.3 Swelling
				D2.4.4 Stomachache

				D2.4.5 Urge to regurgitate
				D2.4.6 Regurgitation
				D2.4.7 Gases
				D2.4.8 Vomits
				D2.4.9 Urge to defecate
				D2.4.10 Left intestinal pain
				D2.4.11 Right intestinal pain
				D2.4.12 Defecation: Normal consistency
				D2.4.13 Defecation: abnormal loose feces
				D2.4.14 Defecation: diarrhea
				D2.4.15 Defecation: bloody feces
				D2.4.16 Nausea
				D2.4.17 Dizziness
				D2.4.18 Acute transient abdominal pain
			D2.5 Gastrointestinal discomfort/problem during the test is related to the consumption of any food, liquid or supplement	D2.5.1Yes
				D2.5.2 No
			D2.6 In case of discomfort, the type of food that causes it En caso de presentar malestar, tipo de alimento que lo ocasiona.	D2.6.1 Examples of foods that can cause gastro-intestinal discomfort/problems
			D2.7 Reasons or reasons why it could have happened	D2.7.1 Explanation and detail of the reasons or reasons why it could have happened

Table S6. Map of codes and coherencies of Planning of the test at the dietary-nutritional level for the proposal for online questionnaire validation for endurance events.

RESEARCH QUESTIONS	THEME	METACODES	CODES	SUBCODES
What questions would you ask to find out about the dietary and nutritional planning prior to a competition?	E. Planning of the competition at the dietary-nutritional level	E1. Questions referring to planning of the dietary-nutritional test from the point of view of expert dietitians-nutritionists	E1.1 Special preparation (training sessions for the gastrointestinal tract, specific diet days before, etc...) for this competition	E1.1.1 Yes
				E1.1.2 No
			E.1.2 Previous planning of the dietary-nutritional strategy for the competition	E1.2.1 Yes
				E1.2.2 No
			E1.3 Nutritional advice received by a professional before the competition	E1.3.1 Dietist-nutricionist
				E1.3.2 Physician/Doctor
				E1.3.3 Coach
				E1.3.4 Nurse
				E1.3.5 Pharmacist
				E1.3.6 Friend/family
				E1.3.7 Web/Internet
				E1.3.8 Physiotherapist
				E1.3.9 None
				E1.3.10 Other
		E2. Questions referring to planning of the dietary-nutritional test from the point of view of athletes	E2.1 Special preparation (training sessions for the gastrointestinal tract, specific diet days before, etc...) for this competition	E2.1.1 Yes
				E2.1.2 No
			E2.2 Previous planning of the dietary-nutritional strategy for the competition	E2.2.1 Yes
				E2.2.2 No
			E2.3 Nutritional advice received by a professional before the competition	E2.3.1 Dietist-nutricionist
				E2.3.2 Physician/Doctor
				E2.3.3 Coach
				E2.3.4 Nurse
				E2.3.5 Pharmacist
				E2.3.6 Friend/family
				E2.3.7 Web/Internet
				E2.3.8 Physiotherapist
				E2.3.9 None
				E2.3.10 Other
